# Characterization and Biomedical Applications of Electrospun PHBV Scaffolds Derived from Organic Residues

**DOI:** 10.3390/ijms26010180

**Published:** 2024-12-28

**Authors:** Anyi Jin, Germán Pérez, Antxon Martínez de Ilarduya, Luis J. del Valle, Jordi Puiggalí

**Affiliations:** 1 Department of Chemical Engineering, Barcelona East School of Engineering (EEBE), Polytechnic University of Catalonia, Av. Eduard Maristany, 10–14, Ed. I2, 08019 Barcelona, Spain; anyi.jin@venvirotech.com; 2 VEnvirotech Biotechnology S.L., Pol. Ind. La Torre del Rector, 08130 Santa Perpetua de la Mogoda, Spain; german.perez@venvirotech.com; 3 Department of Chemical Engineering, Barcelona School of Industrial Engineering (ETSEIB), Polytechnic University of Catalonia, Diagonal, 647, 08028 Barcelona, Spain; antxon.martinez.de.ilarduia@upc.edu; 4 Barcelona Research Center in Multiscale Science and Engineering, Polytechnic University of Catalonia, Av. Eduard Maristany 10–14, 08019 Barcelona, Spain

**Keywords:** poly(3-hydroxybutyrate), poly(3-hydroxybutyrate-*co*-3-hydroxyvalerate), polyhydroxyalkanoates, electrospinning, waste revalorization, biopolymer, bioplastic

## Abstract

This study explores the characterization and application of poly(3-hydroxybutyrate-*co*-3-hydroxyvalerate) (PHBV) synthesized from organic residues, specifically milk and molasses. Six PHBV samples with varying 3-hydroxyvalerate (3HV) content (7%, 15%, and 32%) were analyzed to assess how 3HV composition influences their properties. Comprehensive characterization techniques, including NMR, FTIR, XRD, DSC, TGA, and tensile-stress test, were used to evaluate the molecular structure, thermal properties, crystalline structure, and mechanical behavior. Selected PHBV samples were fabricated into nanofibrous scaffolds via electrospinning, with uniform fibers successfully produced after parameter optimization. The electrospun scaffolds were further analyzed using DSC, GPC, and SEM. Biological evaluations, including cytotoxicity, in vitro drug release, and antibacterial activity tests, were also conducted. The results indicate that the electrospun PHBV scaffolds are biocompatible and exhibit promising properties for biomedical applications such as tissue engineering and drug delivery. This study demonstrates the potential of using organic residues to produce high-value biopolymers with tailored properties for specific applications.

## 1. Introduction

Polyhydroxyalkanoates (PHAs) are a diverse group of biodegradable polyesters naturally synthesized by bacteria through fermentation processes. This family includes over 150 different types, each distinguished by unique carbon side chains and monomer compositions that contribute to a wide range of physical and chemical properties [1,2]. PHAs have garnered significant attention as sustainable alternatives to conventional plastics because of their remarkable biodegradability and biocompatibility [3]. Among these, polyhydroxybutyrate (PHB) and poly(3-hydroxybutyrate-*co*-3-hydroxyvalerate) (PHBV) are the most studied because of their easier accessibility. However, commercially available PHBV typically contains a low 3-hydroxyvalerate (3HV) proportion (<5%). This composition leads to certain structural disadvantages as it considerably increases its properties of stereoregularity, crystallinity, and rigidity [4]. These drawbacks are limiting their applicability especially when flexibility is required.

In response to these limitations, research has demonstrated that enhancing the 3HV content within PHBV can significantly increase its flexibility and toughness [5]. This enhancement occurs as the 3HV units interrupt the chain regularity of PHB, making the polymer more ductile and less rigid. Such modifications not only improve the polymer processability but also expand its range of applications. In the fermentation process, various strategies have been developed to achieve higher 3HV content, for example, using specific microbes capable of producing high 3HV content, altering substrates such as by adding valerate or propionic acid to favor 3HV production, and applying genetic engineering to modify bacteria for desired PHBV synthesis [6,7,8,9]. However, these approaches often lead to increased production costs or involve advances in technology that require significant research and development efforts to make them more cost-effective and scalable for industrial applications.

The use of industrial waste as substrate for PHBV production has been considered as an alternative approach for reducing the overall cost of production. Various industrial by-products, including agricultural residues, food waste, and other organic materials, have been identified as viable substrates for PHBV biosynthesis [10,11,12,13]. In this work, six PHBV samples with varying 3HV contents (7%, 15%, and 32%) derived from two different residues, milk and molasses, are evaluated and compared in terms of molecular structure, thermal properties, crystalline structure, and mechanical behavior. These agricultural and livestock by-products are rich in nutrients essential for bacterial growth and metabolism. Molasses contains a high concentration of sugars, primarily sucrose, which serves as an excellent carbon source for many bacteria, while milk provides lactose, proteins, and other nutrients beneficial for both carbon and nitrogen sourcing in microbial growth and PHA production. The use of these organic residues presents a cost-effective alternative for PHBV production, which reduces the overall cost compared to using pure sugars or other more expensive substrates. Finally, the fabrication of nanofibrous scaffolds with these PHBV samples through the electrospinning technique is explored to evaluate their applicability in the biomedical field.

Electrospinning is a versatile technique that allows the production of ultrafine fibers with high surface area-to-volume ratios and porosity. These attributes are critical for creating scaffolds that mimic the extracellular matrix, thereby promoting cell attachment, proliferation, and differentiation [14,15], which are essential for tissue engineering and other biomedical applications [16]. In this study, the electrospinning process parameters are optimized to achieve uniform fiber morphology. The resulting PHBV scaffolds are characterized using various techniques, including SEM for fiber morphology, DSC for thermal behavior, GPC for assessing polymer degradation, and cytotoxicity tests to ensure biocompatibility. Additionally, in vitro drug release and antibacterial activity assays are conducted to evaluate the potential of these scaffolds for drug delivery applications.

By integrating sustainable PHBV production from organic residues with advanced electrospinning techniques, this research aims to develop cost-effective, high-performance biomaterials suitable for a range of biomedical applications, highlighting a promising approach to environmental sustainability and healthcare innovation.

## 2. Results and Discussion

### 2.1. Structural Analysis of PHBV Samples

#### 2.1.1. FTIR Analysis

**Figure 1:**
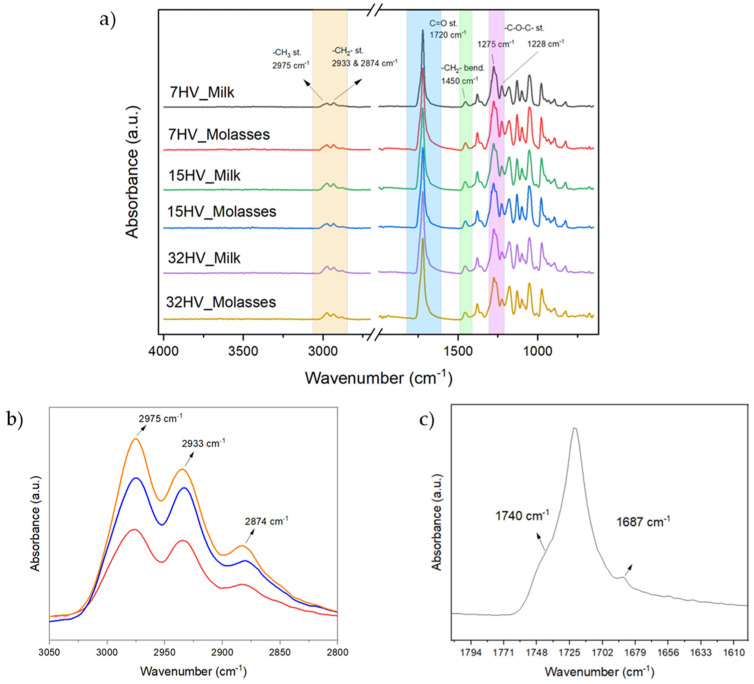
(**a**) FTIR absorption spectra of PHBV derived from molasses and milk with varying 3HV content. (**b**) Expanded section of 3050 to 2800 cm^−1^ of PHBV derived from molasses samples with 7% 3HV (red), 15% 3HV (blue), and 32% 3HV (orange). (**c**) Expanded section of 1800–1610 cm^−1^ of 7% 3HV from milk.

The FTIR spectra of representative PHBV samples (named by their 3HV content and source, e.g., 7HV_Milk corresponds to 7% of 3HV biosynthesized from milk residues) are presented in Figure 1a. Despite the differences in 3HV content (7%, 15%, and 32%) and sources (milk and molasses), the spectra of all samples exhibit similar characteristic bands, confirming the consistent primary chemical structure of PHBV. Key bands include the C=O stretching of the ester group at 1720 cm^−1^ and the C-H stretching vibrations of methylene and methyl groups at 2874 cm^−1^, 2933 cm^−1^, and 2975 cm^−1^, which are consistent with earlier reports [17,18,19,20].

The intensities of the C-H peaks absorbed in the range of 3050–2800 cm^−1^ gradually increased with higher 3HV content, which is expected due to the increase in methylene side groups (Figure 1b). A detailed analysis of the C=O stretching peak reveals that it consists of a main peak and two minor peaks at 1740 cm^−1^ and 1687 cm^−1^ (Figure 1c). These correspond to the C=O vibration of the PHBV amorphous phase and hydrogen bonding interactions between the oxygen atom in the C=O group and the nearest hydrogen atom, respectively, as indicated by Kansiz et al. [18].

Other characteristic peaks include the -CH_2_- bending and -C-O-C stretching, observed at 1228 cm^−1^ and 1450 cm^−1^. Some authors have used these peaks to estimate the crystallinity content of PHBV samples, as these peaks are sensitive to the crystalline phase [21]. However, in this case, due to the complexity of the peak region, only minor variations in peak intensities can be attributed to differences in 3HV content.

#### 2.1.2. XRD Results

**Figure 2:**
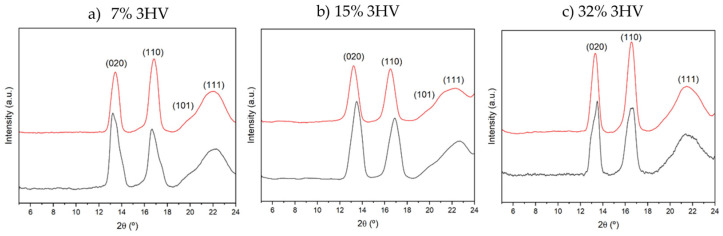
XRD of PHBV samples derived from milk residues (black line) and molasses residues (red line) with the following: (**a**) 7% 3HV; (**b**) 15% 3HV; (**c**) 32% 3HV.

The X-ray diffraction (XRD) patterns of PHBV samples derived from different feedstocks and varying 3HV contents are shown in Figure 2. The diffraction patterns display peaks at 2*Ɵ* values of 13°, 16°, 21°, and 22°, corresponding to the (020), (110), (101), and (111) crystalline planes, respectively [22,23,24]. These reflections indicate the presence of an orthorhombic crystalline lattice with space group P2_1_2_1_2_1_. The corresponding *d*-spacings and lattice parameters *a*, *b*, and *c* are presented in Table 1.

The main trends observed in the studied series are as follows: (a) The peak intensity of the (020) plane is higher than that of the (110) plane in samples derived from milk residues, but this is not the case for samples derived from molasses. (b) The intensity of the (101) peak decreases as the 3HV content increases and also when the polymer is derived from molasses instead of milk. The loss of crystalline order, whether due to high 3HV content or the use of molasses as a feedstock, significantly affects the (101) diffraction peak. (c) The variation in peak intensities suggests that the molecular alignment within the crystalline domains of PHBV favors the (020) plane for milk-derived samples, while it favors the (110) plane for molasses-derived samples. This trend is also reflected in the *d*-spacing and lattice parameters: for samples with the same composition, the *d*_020_ spacing is consistently larger in milk-derived samples compared to molasses-derived samples. (d) The lattice parameters *a* and *c* increase with 3HV content for both milk and molasses-derived samples, with milk-derived samples generally exhibiting slightly larger *a* value. The parameter *b* behaves differently: it decreases with increasing 3HV content in milk-derived samples but increases in molasses-derived samples.

The variations in diffraction peak intensities, lattice parameters, and *d*-spacings reflect differences in molecular arrangement and the degree of crystallinity influenced by the feedstock. The larger intensity of the (020) peak, the larger *d*_020_ spacing, and the higher *a* parameter in milk-derived samples suggest a higher degree of crystallinity or preferred molecular alignment along the (020) plane. The increasing *d*_110_ and *d*_111_ spacings with higher 3HV content for both feedstocks indicate a consistent trend of expanding lattice dimensions.

#### 2.1.3. NMR Analysis

**Figure 3:**
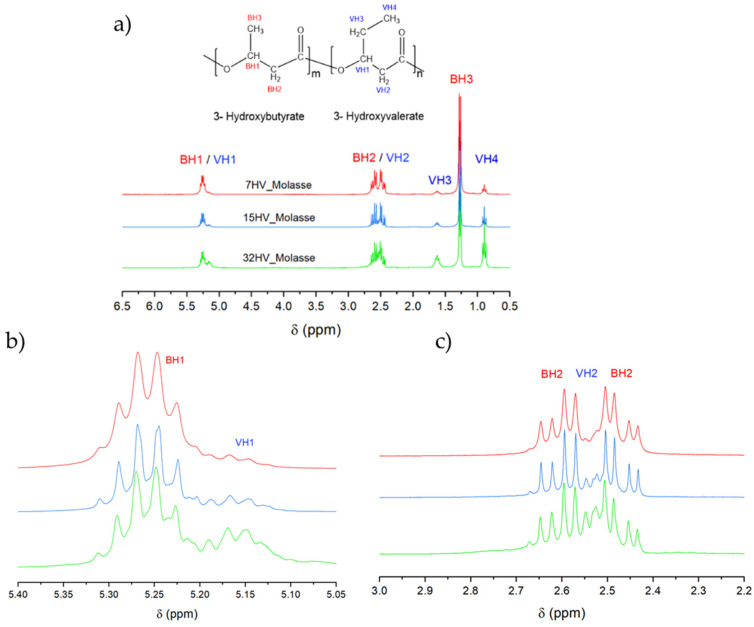
(**a**) ^1^H NMR spectra of PHBV samples derived from molasses with varied 3HV content. (**b**) Expanded signals assigned to BH1/VH1 chemical groups. (**c**) Expanded signals assigned to BH2/VH2 chemical groups.

The ^1^H NMR spectra of PHBV samples with varying 3HV content showed no significant differences between polymers derived from molasses and milk residues. Figure 3a presents the spectra and assignments for the representative series derived from molasses. The B and V refer to the units of 3HB and 3HV, respectively. Due to the similar chemical environments, overlap occurs between the BH1 and VH1 protons (which belong to the CH group) and between the BH2 and VH2 protons (CH_2_ group linked to the carbonyl group). Specifically, the BH1/VH1 protons appear around δ 5.1–5.4 ppm, while the BH2/VH2 protons appear at approximately δ 2.4–2.7 ppm. The spectra reveal that the multiplets attributed to BH2/VH2 and BH1/VH1 become increasingly complex as the 3HV content increases (Figure 3b,c), presumably due to the increased intensity of the VH2 and VH1 signals. Despite the variation in 3HV content, the chemical shifts of the proton peaks remain constant, corroborating the findings reported by Pramanik et al. [20]. Quantification of 3HV content within the PHBV copolymers can be achieved by integrating the area under the peaks corresponding to the methyl side chain of 3HB (BH3) at δ 1.2–1.3 ppm and the methyl side chain of 3HV units (VH4) at δ 0.9–1.1 ppm (Table 2).

**Figure 4:**
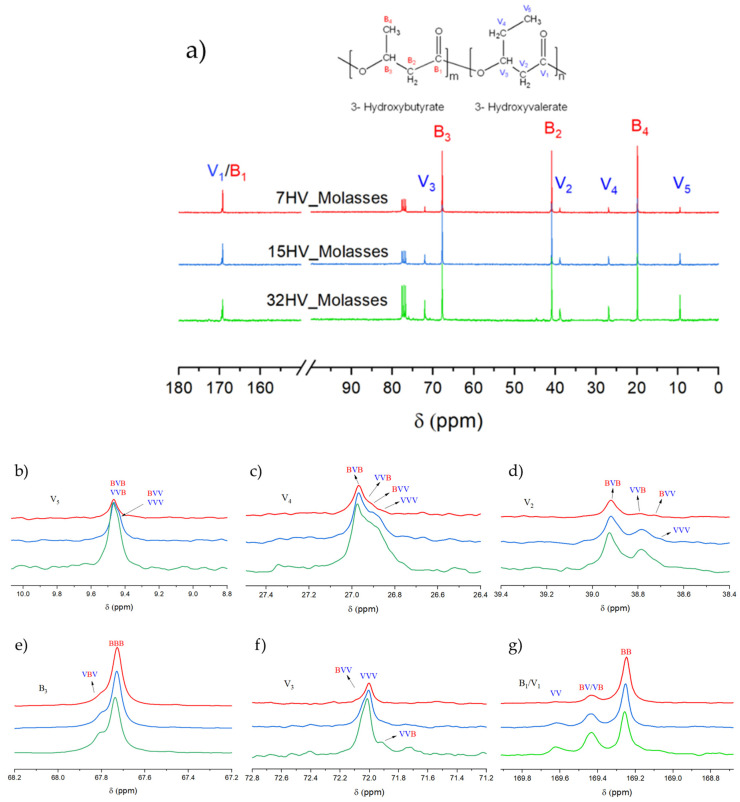
(**a**) ^13^C NMR spectra of PHBV samples derived from molasses residues with varying 3HV content: 7% 3HV (red line), 15% 3HV (blue line), and 32% 3HV (green line). (**b**–**g**) Expanded signals assigned to different chemical groups.

The sequence distribution of PHBV derived from molasses with varying 3HV content was determined using ^13^C NMR spectroscopy. The spectra illustrated in Figure 4a show the chemical composition and magnified signals of each chemical group. According to previous studies, the distribution of monomer units in PHBV can be characterized as either diads (VB/VV) or triads (VBV/BVB/VVV/BBV), where V represents the 3HV unit and B represents the 3HB unit. The assignment of each peak is referenced in prior literature [25,26,27].

The most significant peaks in the ^13^C NMR spectra are shown in detail in Figure 4b–g. The peak around 169 ppm (Figure 4g) corresponds to the carbonyl carbon, which is split into three peaks representing the diads BB, VB/BV, and VV. The areas of these peaks are used to determine the sequence distribution of 3HV and 3HB comonomers by calculating the *D* parameter (*D* = (*F*_VV_ × _BB_)/(*F*_BV_ × *F*_VB_), where *F* represents the relative mole fraction of each diad. According to the literature, copolymers are randomly distributed when *D* is close to 1. *D* values higher than 1 suggest a block-monomer distribution, while values lower than 1 are characteristic of alternating copolymer [25,28].

According to the results shown in Table 2, samples with high 3HV content (32%) and those with low 3HV content (7%) derived from molasses residues are randomly distributed, with *D* values close to 1. However, samples with low 3HV content (7%) derived from milk residues exhibit block distribution, with a *D* value of 2.80. Similarly, both samples with 15% HV content show a tendency for block distribution, with *D* values of 2.67 for molasses-derived samples and 6.32 for milk-derived samples.

Other significant peaks include V2 at δ 38.6–38.8 ppm, V3 at δ 71.6–72.3 ppm, V4 at δ 26.6–27.2 ppm, and V5 at δ 9.2–9.8 ppm, which correspond to the V-centered triads. The intensities of these peaks increase with higher 3HV content in PHBV, as shown in Figure 4b–f. Additional peaks of interest are attributed to the carbons in the side chain and methylene group of 3HB (B2 at δ 41 ppm and B4 at δ 20 ppm). Due to their distinct chemical environments, these peaks do not overlap with 3HV units and appear as single peaks, likely due to the dominance of 3HB content.

### 2.2. Thermal Behavior of PHBV Samples

#### 2.2.1. DSC Calorimetric Data

Typical DSC curves of PHA copolymers are characterized by an unresolved double melting peak (between 144 °C and 173 °C) with distinctive relative intensities. This double peak arises from different lamellar populations, with the lower-temperature peak corresponding to primary thin crystals that can recrystallize into thicker crystals upon heating. The relative intensity between these peaks varies significantly depending on the stability of the primary crystals [29,30]. Several factors can influence the thermal properties of the PHBV series, including molecular weight, composition, and the type of residues used in bacterial fermentation.

**Figure 5:**
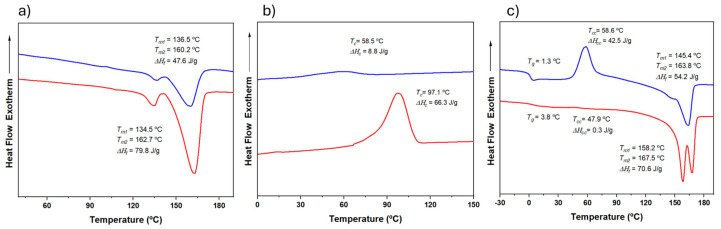
DSC traces corresponding to the first heating (**a**), cooling (**b**), and second heating (**c**) runs of PHBV samples having 15% of 3HV content, which are obtained from milk (red) and molasses (blue) residues.

Figure 5a shows the DSC traces (heating, cooling, and reheating) for a representative copolymer from the series with intermediate 3HV content (15%) obtained from milk and molasses residues. The copolymer derived from molasses was initially less crystalline and exhibited minimal crystallization during cooling (Figure 5b). However, it underwent cold crystallization during the second heating, resulting in a semicrystalline material (Figure 5c). In contrast, the copolymer obtained from milk showed easier crystallization, as evidenced by a crystallization peak detected during cooling and greater enthalpy (i.e., higher crystallinity) in both the first and second heating runs. This behavior is likely due to the block distribution of the copolymer, which enhances its ability to undergo crystallization.

**Figure 6:**
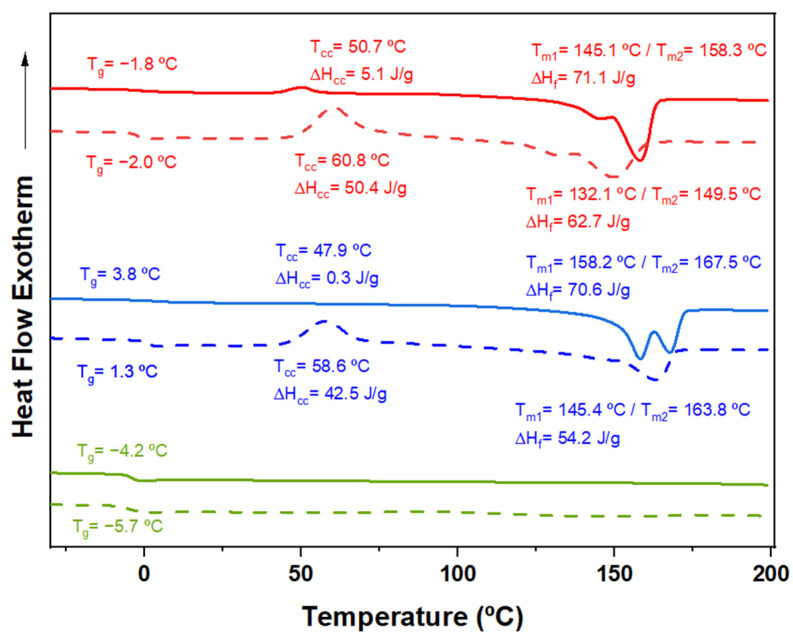
DSC second heating run of PHBV samples having 7% (red), 15% (blue), and 32% (green) of 3HV units. Copolymers were obtained from milk (solid lines) and molasses feedstocks (dashed lines).

Figure 6 compares the second heating trace of samples with varying 3HV content and feedstock. As expected, crystallinity decreases with increasing comonomer content, as the minor 3HV units hinder the crystallization process of the major component. The 3HV units are excluded from the crystalline regions, resulting in thinner crystalline structures with lower melting temperatures and reduced crystallinity [5,31]. However, as summarized in Table S1, the sample with 15% 3HV (15HV_Milk) crystallized more quickly than the sample with 7% 3HV (7HV_Milk), exhibiting crystallization peaks at 97.1 °C and 79.9 °C, and crystallization enthalpies of 66.3 and 58.1 J/g, respectively. This anomaly is likely due to the high block distribution in the 15% 3HV samples (see Table 2), which enhances their ability to crystallize more rapidly despite the higher 3HV content. Additionally, the higher *T_g_* observed for the 15% 3HV samples (3.8 °C and 1.3 °C for samples derived from milk and molasses, respectively) can also be attributed to the restricted mobility of the polymer chains due to the higher molecular weight and block distribution of monomers.

In summary, while the general trend indicates that increasing 3HV content reduces crystallinity and decreases melting and crystallization temperatures, the block distribution in the 15% 3HV sample overrides this trend, leading to enhanced crystallization behavior and higher crystallization and melting temperatures.

#### 2.2.2. Thermal Degradation of PHBV Samples

**Figure 7:**
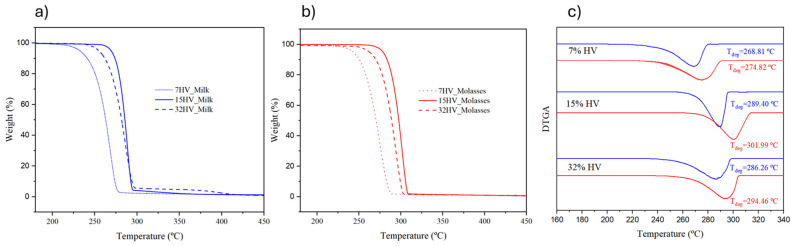
TGA plots of PHBV samples with varied 3HV content, which are derived from milk (**a**) and molasses (**b**) residues; (**c**) DTGA curves of samples derived from milk (blue line) and molasses (red line) residues.

The thermal stability of PHBV samples was assessed using TGA. Summarized data, including the temperatures at 5%, 50%, and 90% weight loss, as well as the residual mass at 310 °C, are presented in Table S2. The TGA curves of percentage weight evolution for PHBV samples with varying 3HV content, separated by feedstocks, are shown in Figure 7a for samples derived from milk residues and Figure 7b for those derived from molasses residues.

In general, the copolymers exhibited moderate thermal stability, with the temperature corresponding to a 5% weight loss ranging from 232 °C to 279 °C. Decomposition consistently occurs in a single step and was completed between 280 °C and 310 °C. Degradation appears to be more pronounced in the 3HB blocks, which is in agreement with the known low thermal stability of the P3HB homopolymer and its difficulty in processing from the melt state. The P3HB homopolymer is inherently thermally unstable due to its susceptibility to random chain scission at elevated temperatures, particularly during melt processing. This instability is linked to its highly crystalline nature, which reduces chain mobility and makes it more prone to thermal degradation.

As the 3HB content increases in PHBV copolymers, the polymer exhibits more characteristics of P3HB, including its lower thermal stability. This trend is evident in the onset decomposition temperatures (or temperatures at 5% weight loss). For example, samples with higher 3HB content exhibit lower onset decomposition temperatures, such as 232 °C for 7HV_Milk compared to 269 °C for 15HV_Milk. The lower onset temperature in 7HV_Milk indicates earlier degradation, consistent with the higher fraction of thermally unstable 3HB blocks.

Molecular weight also affects thermal stability, with enhanced decomposition detected in samples with lower molecular weights. This is evident when comparing the thermal behavior of samples with 15% and 32% 3HV units, where the latter, despite its higher 3HV content, exhibited lower thermal stability due to its reduced molecular weight.

As shown in Figure 7, samples derived from molasses residues exhibited greater thermal stability (as indicated by higher degradation peaks in the DTGA plots) than their milk-derived counterparts. The reduced thermal stability of the milk-derived samples may be attributed to the lower stability of the 3HB blocks, combined with their blocky sequence distribution (Section 2.1.3), which can increase structural irregularities and enhance susceptibility to thermal degradation.

Furthermore, the residual weight percentages at 310 °C ranged from 2.1% to 5.3% for milk-derived samples, while molasses-derived samples exhibited consistently lower residual weights, ranging from 1.4% to 1.8%. These residues are likely due to impurities from insufficient purification during upstream processing, which may accelerate the thermal degradation of PHBV samples.

### 2.3. Mechanical Properties of the PHBV Samples

**Figure 8:**
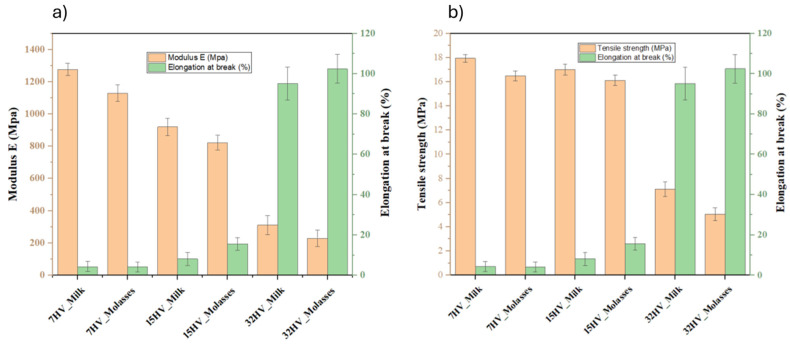
Mechanical properties of PHBV film samples derived from different residues: (**a**) Plot of the change in modulus and elongation; (**b**) Plot of the change in tensile strength and elongation.

The mechanical properties of PHBV samples derived from different sources with varying 3HV content were evaluated using film samples prepared by solvent casting. As shown in Table 3 and Figure 8, the 3HV content directly influences the mechanical performance of PHBV. Specifically, increasing the 3HV content reduces the modulus from 1277 MPa to 230 MPa and the tensile strength from 18 MPa to 5 MPa, while elongation increases from 4% to 102%. This behavior indicates that higher 3HV content makes the samples less stiff and more ductile, as it increases the amorphous regions within the polymer matrix. This trend is consistently observed across both milk- and molasses-derived samples, demonstrating the tunability of PHBV properties through compositional adjustments.

When comparing PHBV samples derived from molasses and milk at the same composition, those from milk exhibit superior mechanical performance. The result aligns with the higher crystallinity observed in milk-derived samples, which is a consequence of their blocky structure.

### 2.4. PHBV Electrospun Fibers

#### 2.4.1. Electrospinning Process Optimization

**Figure 9:**
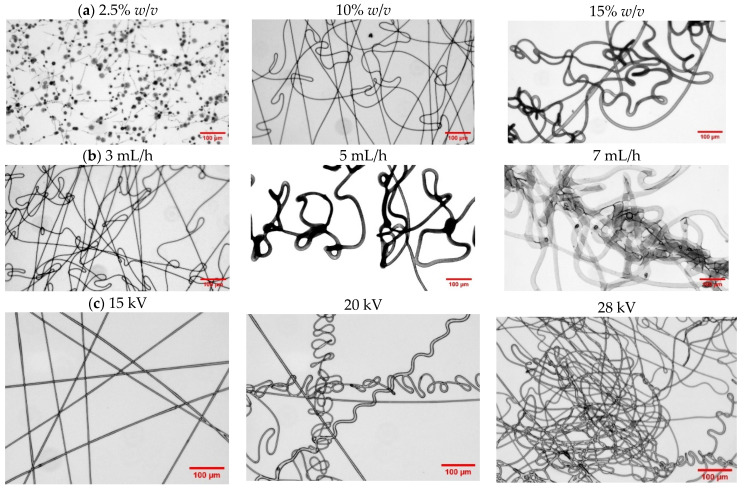
Optical micrographs of electrospun fibers from 7HV_Molasses and processed under different conditions: (**a**) Variation in concentration maintaining feeding rate at 2 mL/h and applied voltage at 15 kV; (**b**) Variation in feeding rate maintaining concentration at 10% *w*/*v* and feeding rate at 2 mL/h; (**c**) Variation in applied voltage maintaining feeding rate at 2 mL/h and concentration at 10% *w*/*v*. Scale bar 100 µm.

The successful formation of PHBV fibrous scaffolds depends on key parameters, such as polymer solution concentration, feeding rate, and applied voltage [32,33,34,35,36]. These parameters were systematically optimized to produce well-defined microfibers with uniform size and minimal defects. The optimized parameters for each PHBV sample are presented in Table 4, while Figure 9 illustrates the influence of these parameters on a representative sample (7HV_Molasses).

According to the results, solution concentration was identified as the most critical parameter influencing fiber formation. Insufficient concentration often results in low solution viscosity, leading to the formation of beaded fibers or even failure to form fibers entirely. As shown in Figure 9a, uniform fibers were only achieved at higher solution concentrations (e.g., 10% *w*/*v* and 15% *w*/*v*). The 10% *w*/*v* concentration resulted in optimal fiber formation, while 15% *w*/*v* led to the formation of thicker fibers. It is important to note that viscosity plays a crucial role in successful fiber formation, as it directly influences the rate of solvent evaporation, which is a key factor in the electrospinning process.

In general, the molecular weight of the polymer also affects viscosity, with higher molecular weight polymers typically forming more viscous solutions. However, in this study, both 15HV_milk and 15HV_molasses samples formed uniform, bead-free fibers at a relatively high solution concentration (15% *w*/*v*). This observation suggests that, while molecular weight contributes to solution viscosity, the inherent nature of the polymer (e.g., crystallinity and 3HV content) plays a more significant role in determining the fiber-forming properties of the solutions. For example, samples with 32% 3HV content, which are more amorphous, required a higher concentration (20% *w*/*v*) to produce good-quality fibers.

Feeding rate also significantly influences fiber morphology. As illustrated in Figure 9b, increasing the feeding rate from 3 mL/h to 7 mL/h resulted in thicker and less uniform fibers, likely due to insufficient solvent evaporation at higher flow rates. The optimized feeding rate of 2 mL/h is demonstrated in Figure 9a at a solution concentration of 10% *w*/*v*.

Similarly, applied voltage affects both the stretching of the polymer jet and the rate of solvent evaporation. As shown in Figure 9c, increasing the voltage from 15 kV to 28 kV resulted in changes to fiber morphology, with excessively high voltages leading to irregular fibers due to instabilities in the jet formation process.

#### 2.4.2. Degradation During the Electrospinning Process

**Figure 10:**
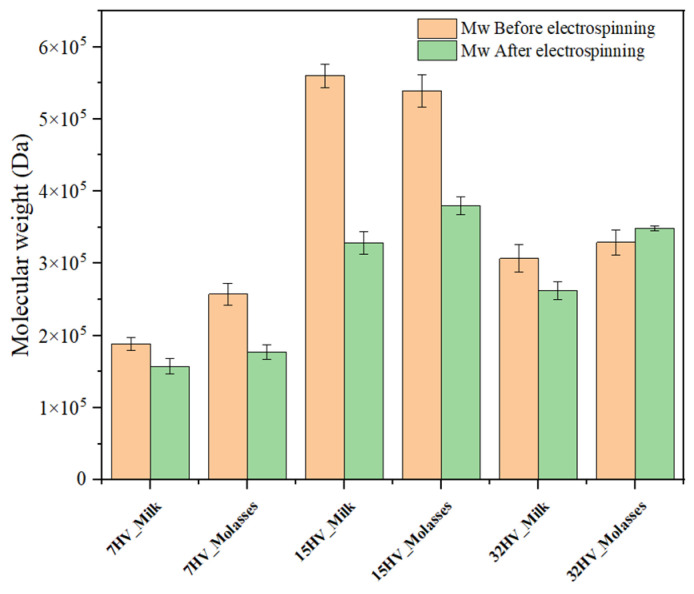
Molecular weight of pristine PHBV and corresponding electrospun fibers.

The variation in molecular weight of PHBV samples after scaffold formation via electrospinning was analyzed using GPC. The results illustrated in Figure 10 indicate no significant change for samples with lower molecular weights (200–300 kDa), with reductions being less than 10%. However, samples with higher molecular weights (above 500 kDa, specifically 15HV_Milk and 15HV_Molasses) exhibited a substantial decrease in molecular weight after the electrospinning process, with a reduction of nearly 40% in both cases.

The observed instability can be attributed to several factors. Higher molecular weight polymers have increased viscosity, which subjects them to greater shear stress during electrospinning, potentially leading to chain scission. Additionally, the electrospinning process itself involves high voltage and rapid solvent evaporation, creating thermal and mechanical stresses that make longer polymer chains more prone to breakage. The possibility of degradation during sample preparation (temperature, time, and agitation) was previously discarded to ensure that the observed molecular weight changes are specifically associated with the electrospinning process.

#### 2.4.3. Morphology of PHBV Electrospun Fibers

**Figure 11:**
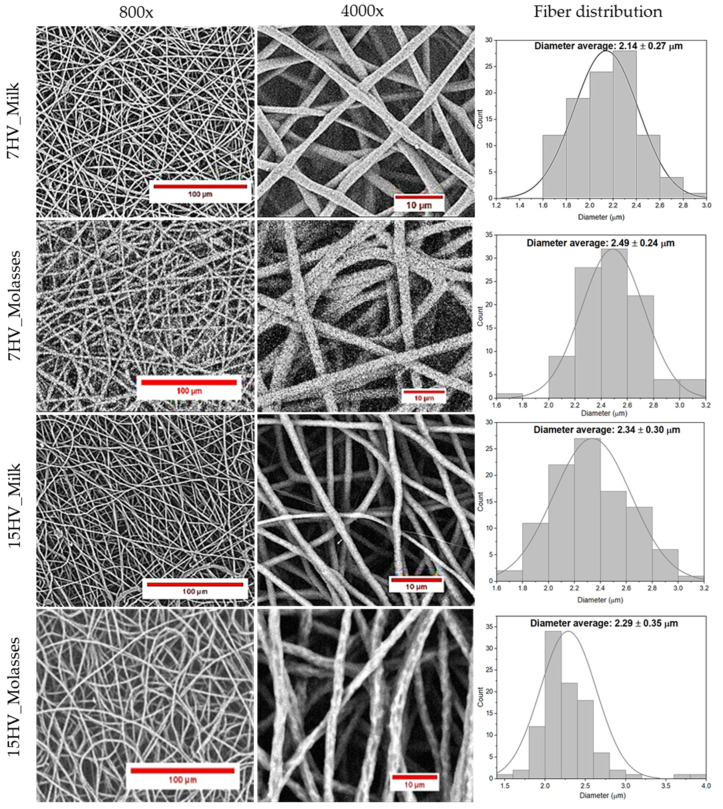

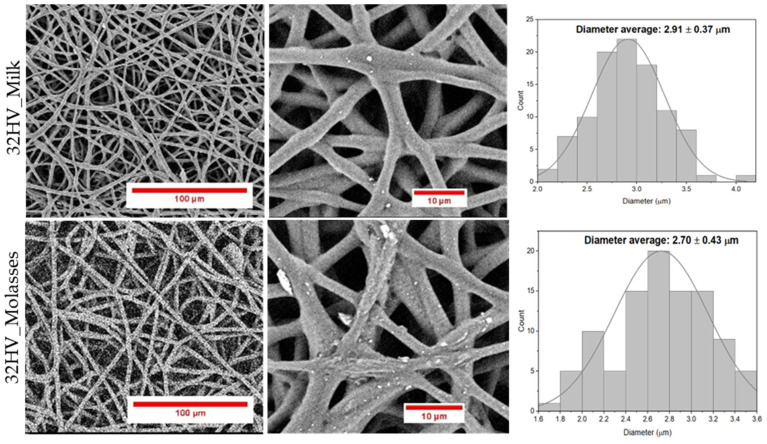
SEM images of the different PHBV electrospun fibers produced under optimized condition and corresponding fiber distribution.

The fiber morphology and diameter size were evaluated using SEM, as shown in Figure 11. The results demonstrated that all PHBV samples successfully produced electrospun fibers with diameters around 2–3 microns, consistent with numerous studies on PHBV electrospun fibers [37,38,39].

However, the choice of solvent plays a crucial role in determining the morphology and size of electrospun fibers, as it directly influences the solution properties, such as viscosity, conductivity, and the rate of solvent evaporation during electrospinning. For instance, chloroform, which was used in this study, tends to produce fibers with larger diameters due to slower solvent evaporation and reduced stretching of the polymer jet under the applied electric field. In contrast, solvent mixtures like DMF/chloroform are known to reduce fiber diameters. HFIP, for example, enhances polymer chain stretching due to its higher polarity and faster evaporation rate, while DMF improves conductivity and facilitates more uniform jet elongation. As a result, these solvents or mixtures are often employed to produce finer fibers with average diameters below 1 micron [40,41,42,43,44].

Additionally, the SEM images (Figure 11) revealed differences in surface texture between milk-derived and molasses-derived fibers. For example, 15HV_Molasses exhibited more rugged and irregular surfaces compared to the smoother surface of 15HV_Milk. This disparity can be attributed to differences in crystallinity between samples. As noted earlier, molasses-derived samples exhibit lower crystallinity due to their more random sequence distribution of 3HV units, which may lead to localized inconsistencies during the electrospinning process. These inconstancies can result in uneven solvent evaporation and solidification of the polymer jet, ultimately producing the observed rougher fiber surface.

#### 2.4.4. Thermal Behavior of PHBV Electrospun Fibers

**Figure 12:**
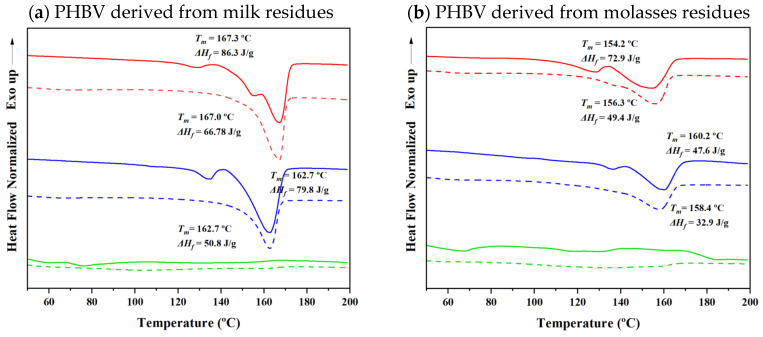
First DSC heating curve of virgin PHBV samples derived from milk residues (**a**) and molasses residues (**b**) with 7% 3HV (red), 15% 3HV (blue), and 32% 3HV (green); the corresponding electrospun fibers are illustrated with dashed lines.

Figure 12 represents DSC profiles obtained from the first heating cycle of PHBV samples derived from different residues with varying 3HV content, both before electrospinning and after forming electrospun fibers. The profiles illustrate notable differences in the thermal behavior of the samples, highlighting changes in crystallinity, melting temperatures, and peak complexity.

The DSC profiles of the electrospun fibers (shown with dashed lines) display lower complexity in their melting peaks compared to the original samples. This reduced complexity suggests that thicker crystals are directly formed during the electrospinning process. The alignment and rapid solidification of fibers likely facilitate the creation of more uniform crystalline structures, resulting in simpler and fewer peaks. Additionally, the electrospun fibers exhibit a slightly higher melting temperature, which is consistent with the presence of thicker crystals. Thicker crystalline regions are more thermally stable, requiring higher temperatures to melt.

Despite the improved alignment achieved through electrospinning, the DSC profiles indicate lower crystallinity in the electrospun fibers, with almost 20 J/g of enthalpy (∆*H_f_*) reduced in each case. The reduction in crystallinity suggests that the electrospinning process hinders crystal formation, potentially due to the rapid solvent evaporation during fiber formation. The lack of sufficient time for the polymer chains to arrange into well-ordered crystalline states may hinder full crystallization, leading to an overall reduction in crystallinity compared to the original samples.

### 2.5. In Vitro Drug Release of PHBV Scaffolds

One of the key features of PHBV is its biocompatibility, making it suitable for biomedical applications [14,45], such as a release drug system. In this study, PHBV samples derived from molasses, with varying 3HV content, were selected to evaluate the incorporation of chloramphenicol (CAM) into scaffolds via electrospinning. The same process parameters presented in Table 4 were used to produce CAM-loaded scaffolds. Samples from milk residues were excluded due to the high residue content detected in TGA analysis (Section 2.2.2). CAM was chosen for its antibacterial activity and because it has recently been suggested as a potential antitumor agent [46].

**Figure 13:**
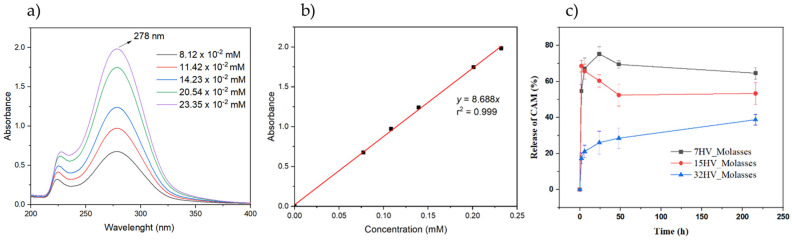
(**a**) UV−spectra of CAM solutions at the indicated concentrations. The spectra showed the absorption band of the *p*−nitrophenyl chromophore. (**b**) Calibration curve for absorbance measurements at 278 nm in PBS medium. (**c**) In vitro release of CAM from PHBV electrospun scaffold with different contents of 3HV units.

The UV spectra of CAM in PBS showed a characteristic band at 278 nm, which correlated with CAM concentration (Figure 13a) to quantify the antibacterial drug in the release experiments. A calibration equation (y = 8.688x) with a linear regression coefficient (r = 0.999) was established (Figure 13b). Three PHBV scaffolds loaded with CAM were immersed in buffer phosphate saline (PBS, pH of 7.2–7.4), and CAM release was monitored over time. The concentration of CAM released, calculated using the calibration curve, was expressed as a percentage (Figure 13c). The PHBV scaffolds with low 3HV content released about 70–80% of CAM within one day, while scaffolds with the highest 3HV content released only 40% after nine days. PHBV with 15% of 3HV reached nearly 50–70% release within one day. In addition, the rapid initial release in the form of a burst stands out when the samples contain 7% or 15% 3HV; this release may be due to a low interaction between the drug and the polymer matrix.

These results suggest that CAM release is influenced by PHBV composition, with faster release from scaffolds with lower 3HV content. Conversely, a steady, prolonged release can be achieved with PHBV containing higher 3HV content, allowing for a tailored release profile. The prolonged release may be related to the decrease in crystallinity, and consequently, the interaction of CAM with the amorphous phase of the polymer matrix could be favored [47,48,49,50].

### 2.6. Antibacterial Activity of PHBV Scaffolds

**Figure 14:**
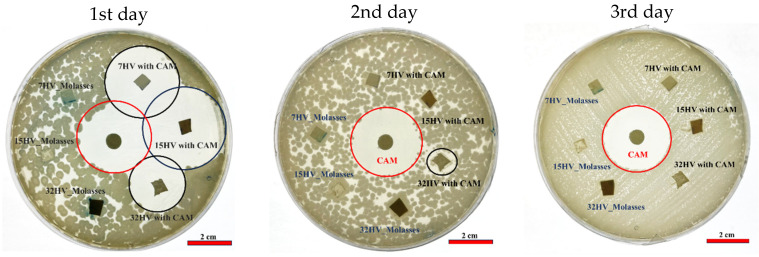
*S. aureus* growth inhibition exposed to CAM-loaded PHBV scaffolds and reapplied in consecutive days.

**Figure 15:**
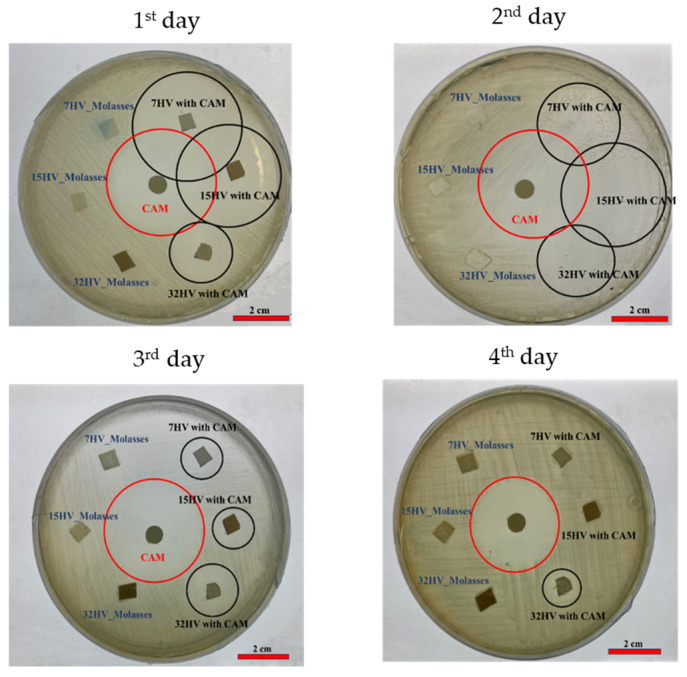
*E. coli* growth inhibition exposed to CAM-loaded PHBV scaffolds and reapplied in consecutive days.

The antibacterial efficacy of CAM-loaded PHBV scaffolds was tested against Gram-negative (*E. coli*) and Gram-positive (*S. aureus*) bacteria after 24 h of incubation. PHBV scaffolds without CAM served as references, while CAM-impregnated disks acted as controls. Clear halos of dead cells around both CAM-loaded scaffolds and CAM disks (Figure 14 and Figure 15) confirmed effective antibacterial activity.

In agar plates seeded with *S. aureus* (Figure 14), samples with lower 3HV content released CAM rapidly on the first day. Only the 32% 3HV sample remained active on the second day, as indicated by a small halo. A similar pattern was observed for *E. coli* (Figure 15), where most of the drug was released on the first day, creating a prominent halo of inhibition of bacterial growth. By the second day, the halo size around the sample with 7% 3HV had decreased significantly, indicating a substantial drop in CAM concentration. The 15% 3HV sample showed sustained antibacterial activity on the second day but was inactive by the third. In contrast, the 32% 3HV sample exhibited a more prolonged release, with antibacterial effects persisting until the fourth day.

Overall, CAM was less effective against *S. aureus* than *E. coli* since no activity was observed on the second and third days. This feature suggests that a higher CAM concentration is needed to achieve bactericidal effects. These results align with the in vitro drug release tests shown in Figure 13c, where higher 3HV content in PHBV scaffolds corresponded to slower CAM release compared to scaffolds with lower 3HV content.

### 2.7. Cytotoxicity Evaluation of PHBV Scaffolds

**Figure 16:**
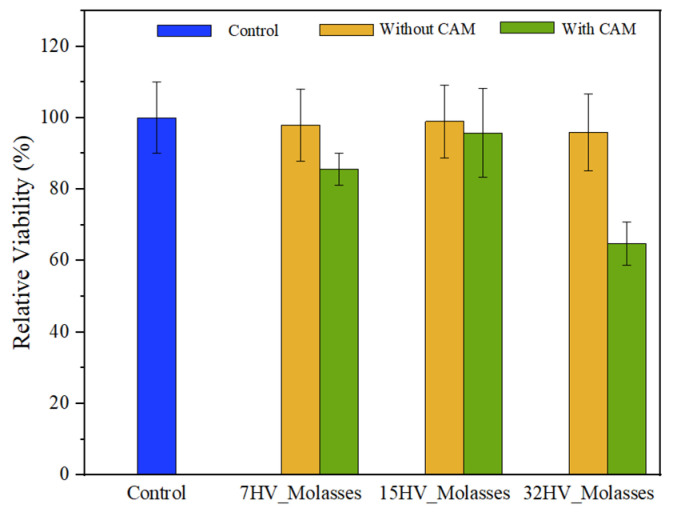
Viability of COS-1 cell on PHBV electrospun scaffolds with and without CAM loaded.

The cytotoxicity of PHBV scaffolds, with and without CAM loading, was evaluated using a COS-1 cell growth assay for 24 h. Figure 16 shows that COS-1 cells remained viable on PHBV scaffolds without CAM, indicating no cytotoxic effect.

However, a significant reduction in cell viability was observed in CAM-loaded scaffolds with high 3HV content (32%), likely due to the presence of CAM within the fiber matrix. In this study, PHBV electrospun fibers were loaded with a dose corresponding to the in vitro LD_50_ of CAM (2.5 M–4 M) [51]. Thus, the results obtained with the 32% HV sample are consistent with CAM loading performed. In contrast, scaffolds with lower 3HV content showed less inhibition of cell viability as CAM was released more rapidly into the medium during the conditioning of samples for culture (e.g., incubation in culture medium for 30 min; see Section 3).

Overall, these findings suggest that while PHBV scaffolds are inherently biocompatible, the presence of the drug can affect cell adhesion, especially in scaffolds with higher 3HV content. The controlled release of CAM from these scaffolds can be beneficial for applications requiring sustained drug delivery, although it may temporarily affect cell viability and adhesion.

## 3. Materials and Methods

### 3.1. PHBV Samples

Poly(3-hydroxybutyrate-*co*-3-hydroxyvalerate) (PHBV) samples, derived from milk and molasses residues with varying 3HV content, were provided by Venvirotech Biotechnology S.L. (Catalonia, Spain). The residues were subjected to acidogenic fermentation to produce volatile fatty acids, which were subsequently used as feedstock for a secondary microbial fermentation process employing mixed bacterial cultures sourced from a wastewater treatment plant. The mixed cultures underwent a feast-famine regime to selectively enrich bacterial populations capable of synthesizing PHBV.

Chloroform (CHCl_3_) and formic acid, used as solvents, were purchased from Sigma Aldrich (St. Louis, MO, USA). Chloramphenicol (CAM) used for drug delivery tests was also purchased from Sigma Aldrich (St. Louis, MO, USA). All chemicals were used as received without further purification.

### 3.2. Characterization of PHBV Samples

#### 3.2.1. Molar Mass Determination

The molecular weight of the original powder samples and electrospun fiber samples was determined by Gel Permeation Chromatography (GPC) using a liquid chromatographic pump (Shimadzu, model LC-8A, Tokyo, Japan) controlled by the LC Solution software version 1.21 (Shimadzu, Tokyo, Japan). The polymer was dissolved in chloroform and eluted in 1,1,1,3,3,3-hexafluoroisopropanol (HFIP) containing sodium trifluoroacetate (CF_3_COONa) at a concentration of 0.05 M. The flow rate was set to 1 mL/min, with an injection volume of 20 µL and a sample concentration of 10 mg/mL. A PL-HFIP-gel column (Agilent Technologies Deutschland GmbH, Böblingen, Germany) and a refractive index detector (Shimadzu, model RID-20A, Tokyo, Japan) were used. The number-average and weight-average molecular weights were determined using polymethyl methacrylate standards.

#### 3.2.2. FTIR Spectral Analysis

Infrared absorption spectra were recorded at a resolution of 4 cm^−1^, with a Fourier Transform FTIR 4700 Jasco spectrometer (Tokyo, Japan) equipped with a Specac MKII Golden Gate Single Reflection Diamond ATR system. PHBV samples were analyzed by Fourier-transform infrared (FTIR) spectroscopy to obtain information regarding its chemical structure.

#### 3.2.3. NMR Spectroscopy

Nuclear magnetic resonance spectroscopy (NMR) was used to determine the chemical composition of PHBV, the ratio of 3-hydroxybutyrate (3HB) to 3-hydroxyvalerate (3HV) units, and the sequence distribution of these units along the polymer chain. NMR spectra were recorded using a Bruker NMR Ascend spectrometer (Billerica, MA, USA) operating at a magnetic field strength of 400 MHz. Approximately 10 mg of the corresponding sample were dissolved in deuterated chloroform. Tetramethylsilane (TMS) was used as an internal reference. All NMR data were analyzed and processed with MestReNova, version 14.3.3.

#### 3.2.4. Thermal Analysis

Differential Scanning Calorimetry (DSC) was conducted using a TA Instruments Q100 series equipped with a refrigerated cooling system (TA Instruments, New Castle, DE, USA), operating over a temperature range of −90 to 550 °C. Experiments were performed under a flow of dry nitrogen, with a sample mass of approximately 4 mg. The instrument was calibrated for both temperature and heat of fusion using an indium standard. Thermal characterization followed a three-step protocol: an initial heating run from room temperature to 200 °C at a rate of 10 °C/min to eliminate thermal history, followed by a cooling run from 200 °C to −50 °C at the same rate, and finally, a second heating run to 200 °C at 10 °C/min. From this second heating run, the melting temperature and the apparent enthalpy of fusion (Δ*H_f_*) were determined. Based on the fusion enthalpy, the relative crystallinity of the crystallized membraned was calculated using the following equation:

(1) $$X(\%)=\left(\frac{{∆H}_{f}}{{∆H}_{ref}}\right)\times 100\%$$

where Δ*H_ref_* represents the enthalpy of fusion for a 100% crystallized PHB polymer, which is 146 J/g as reported in the literature [51,52,53]. Since the 3HV content is significantly lower than the 3HB content (less than 32%), the enthalpy contribution from crystallization is considered only for the HB units.

Thermogravimetric analysis (TGA) data were collected using a Q50 thermogravimetric analyzer (TA Instruments, New Castle, DE, USA) under a flow of dry nitrogen, with sample masses of approximately 5 mg and at a heating rate of 10 °C/min.

#### 3.2.5. Mechanical Analysis

The mechanical properties of the PHBV samples were evaluated by stress-strain tests performed on an Instron Universal Testing Machine (Model 34SC-5 Single Column, Instron Corp., Norwood, MA, USA). The samples were first dissolved in chloroform and subsequently subjected to air evaporation to form uniform thin films. These films were cut into rectangular specimens with dimensions of 100 mm × 25 mm × 0.1 mm for testing. The crosshead rate and load cell capacity were set to 10 mm/min and 5 kN, respectively. Parameters were recorded using Instron software version 4.37. Each test was repeated five times to ensure measurement accuracy.

### 3.3. Fabrication of PHBV Electrospun Fibers

PHBV derived from milk and molasses residues with varying 3HV content were used to fabricate electrospun fibers. The electrospinning process began by dissolving PHBV in chloroform, with gentle agitation and heating to 50 °C to ensure complete polymer dissolution. Formic acid was then added at a concentration of 0.2% *w*/*v* to enhance the conductivity of the solution, facilitating the formation of finer fibers and improving their overall quality. It is important to note that both the agitation time and the solution concentration (Table 4) were adjusted based on the specific PHBV composition to ensure smooth processing and prevent spinneret clogging. Although the optimal viscosity for fiber formation was not evaluated in this study, previous research indicates that this parameter is crucial for the successful processing of polymer nanofibers via electrospinning [54,55]. Inadequate viscosity can alter the solution’s electrical conductivity, leading to bead formation or irregular fibers.

**Figure 17:**
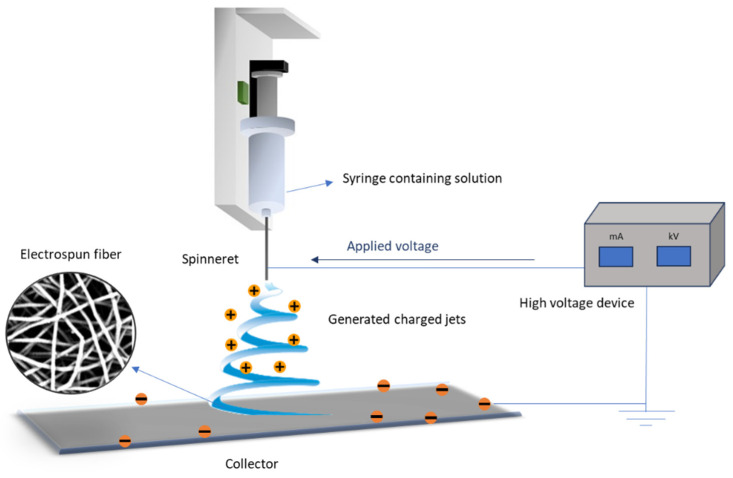
General setup of electrospinning apparatus.

The prepared spinning solution was then transferred into a 2 mL syringe equipped with a 22 G plain metallic-tip BD spinneret. The setup of the electrospinning process is illustrated in Figure 17, which depicts the critical components required for fiber formation from the polymer solution. A voltage was applied between the spinneret and the collector, inducing a charge in the polymer solution that resulted in the formation of charged jets. As these jets traveled toward the collector, solvent evaporation occurred, stretching the solution into fine fibers, which were deposited onto the collector in a random orientation. The collector, covered with aluminum foil, was positioned at a fixed distance of 15 cm from the spinneret. The production of electrospun fibers can be fine-tuned through appropriate optimization of selected process parameters (i.e., feeding rate, solution concentration, and applied voltage) [33].

### 3.4. Morphological and Physical Characterization of Scaffolds

#### 3.4.1. SEM Analysis of PHBV Electrospun Fibers

The morphology of electrospun fibers was characterized using Scanning Electron Microscopy (SEM) using a Focused Ion Beam Neon 40 SEM (Carl Zeiss AG, Oberkochen, Germany) equipped with an energy dispersive X-ray (EDX) spectroscopy system and operating at 5 kV. The samples were coated by carbon sputtering using a K950X Turbo Evaporator (Fedelco S.L., Madrid, España). This technique provided a detailed visualization of the physical properties of the scaffolds, including the uniformity of fiber distribution, the diameter of fibers, the presence of beads or irregularities, and the overall quality of the microstructure of the scaffolds. The SEM images of the scaffolds were captured at various magnifications. The diameters of a significant number of fibers (100 fibers) were measured using Image J software version 1.8.0 to obtain a statistical understanding of the fiber size distribution.

#### 3.4.2. XRD Analysis of PHBV Electrospun Fibers

X-ray Diffraction (XRD) was used to analyze the crystalline structure of the scaffolds. The changes in crystallinity were compared between samples derived from milk and molasses, and the variation in PHBV crystallinity based on composition was investigated in detail. XRD analyses were performed using a Bruker D8 Advance diffractometer (Bruker AXS GmbH, Karlsruhe, Germany). The samples were analyzed at room temperature in reflection mode with incident CuKα radiation (λ = 1.5405 Å), using a step size of 0.05° and an acquisition time of 5 s per step. The unit cell parameters of the polymer were estimated from the characteristic reflections of PHBV (i.e., (020), (110), (101), and (111)) in the 2*θ* range of 5° to 40°.

### 3.5. Biological Studies of PHBV Electrospun Fibers

#### 3.5.1. In Vitro Drug Release from PHBV Electrospun Fibers

Three PHBV samples with varying 3HV content were selected for loading chloramphenicol (CAM), which is used as both an antibiotic and an anticancer agent. To this end, CAM was incorporated into the electrospinning solution at a concentration of 0.2% *w*/*v*. Controlled release experiments were conducted using PHBV electrospun scaffolds cut into square pieces with dimensions of 20 mm × 20 mm × 0.1 mm. The samples were weighed and placed at 37 °C on an orbital shaker at 100 rpm in vessels containing 10 mL of phosphate-buffered saline (PBS, pH 7.2–7.4), which served as the release medium to simulate physiological conditions. Drug concentration in the release medium was evaluated by UV-Vis spectroscopy using a UV-Vis 3600 spectrophotometer (Shimadzu, Kyoto, Japan) with a wavelength range of 200–600 nm and a resolution of 0.5 nm. Calibration curves were generated by plotting absorbance at the corresponding wavelengths against drug concentration. A 1 mL sample was drawn from the release medium at 2 h, 4 h, 6 h, 8 h, 24 h, 3 days, and 1 week. All drug release tests were performed in triplicate to ensure release homogeneity, and the results were normalized by the weight fraction of CAM in the samples and finally averaged.

#### 3.5.2. Antibacterial Activity

The antibacterial activity of the PHBV scaffolds was assessed using a bacterial growth inhibition assay, which measures the formation of inhibition zones. The Gram-negative bacterium *Escherichia coli* (*E. coli*) and the Gram-positive bacterium *Staphylococcus aureus (S. aureus*) were selected for the assay. These bacteria were separately spread on Luria-Bertani (LB) agar plates (Scharlau, Spain). PHBV electrospun scaffolds, both with and without CAM loading, were cut into disks with a diameter of 13 mm and sterilized under UV light for 15 min. A paper disk soaked in CAM solution was used as the control. The loaded disks and the electrospun samples were carefully placed on the surface of the agar plates inoculated with bacteria. The inhibition zones were measured after 24 h of incubation. The same disks of electrospun samples were then transferred to fresh LB agar plates, and the process was repeated until no further inhibition zone was observed, indicating that all CAM had been effectively released. The presence of inhibition zones was interpreted as evidence of effective antibacterial activity.

#### 3.5.3. Cytotoxicity Evaluation

COS-1 cells were cultured in Dulbecco’s Modified Eagle’s Medium (DMEM) containing 4500 mg/L glucose, 110 mg/L sodium pyruvate, and 2 mM *L*-glutamine. The medium was supplemented with 10% fetal bovine serum (FBS), 50 U/mL penicillin, 50 mg/mL streptomycin, and 2 mM *L*-glutamine, and incubated at 37 °C in a humidified atmosphere of 5% CO_2_ and 95% air. The culture medium was replaced every two days. For sub-culturing, cell monolayers were rinsed with PBS and detached by incubating them with 0.25% trypsin/EDTA for 2–5 min at 37 °C. The incubation was stopped by resuspending the cells in 5 mL of fresh medium, and cell concentration was determined by counting with a Neubauer chamber using 4% trypan blue as a vital dye.

For the cell biocompatibility assays, square pieces (10 mm × 10 mm × 0.1 mm) of PHBV electrospun samples, both with and without drug loading, were cut. These pieces were placed in the wells of a multi-well culture plate and sterilized with UV light in a laminar flow cabinet for 15 min. A small amount of silicone adhesive (SilbioneVR MED ADH 4300 RTV, Bluestar Silicones France SAS, Lyon, France) was used to secure the samples in the wells. The samples were incubated in 1 mL of culture medium under standard conditions for 30 min. Afterward, the medium was aspirated, and the material was evaluated for cell adhesion.

The aliquots of 50–100 µL containing 5 × 10^4^ cells were seeded into the wells containing the electrospun scaffolds. The plate was incubated under standard conditions for 30 min to promote cell attachment to the scaffold surfaces. After this period, 1 mL of culture medium was added to each well, and the plate was incubated for an additional 24 h period. Finally, cell viability was determined using the MTT assay.

## 4. Conclusions

This study comprehensively evaluated and compared the physical, thermal, and mechanical properties of PHBV derived from milk and molasses residues. The results demonstrated that the source of PHBV significantly influences its thermal behavior, with milk-derived samples exhibiting higher crystallinity and melting temperatures than those derived from molasses. NMR analysis revealed differences in monomer distribution, which favors a more random arrangement in molasses-derived samples. Minor variations in the X-ray diffraction patterns between samples from both sources suggest differences not only in crystallization behavior but also in crystalline structure. The mechanical and thermal properties of PHBV were also influenced by 3HV content and copolymer distribution, with higher 3HV content leading to increased flexibility and reduced melting temperatures, indicating the tunability of PHBV properties through compositional adjustments.

PHBVs from both milk and molasses were successfully used to produce electrospun fibers with an average diameter of about 2 µm. The antibiotic drug CAM was effectively loaded onto PHBV electrospun scaffolds, and release studies showed that CAM was delivered more rapidly from scaffolds with lower 3HV content. The antibacterial activity of CAM-loaded PHBV scaffolds confirmed that the material did not compromise the efficacy of CAM and maintained effective antibacterial performance. Furthermore, cytotoxicity tests using COS-1 cells demonstrated the inherent biocompatibility of PHBV, with no adverse effects on cell viability observed for scaffolds that were without CAM.

Overall, the findings of this study underscore the potential of PHBV as a versatile biomaterial capable of being tailored through compositional and processing adjustments to meet specific application needs, particularly in the biomedical fields that require controlled drug release and biocompatibility. Additionally, the use of PHBV derived from urban residues highlights its potential contribution to circular economy models by converting waste streams into valuable biomaterials.

## Figures and Tables

**Table 1:** Crystalline lattice parameters *a*, *b*, and *c* calculated from WAXS patterns.

Samples	*d*_(020)_	*d*_(220)_	*d*_(111)_	*a* (nm)	*b* (nm)	*c* (nm)
7HV_Milk	0.670	0.531	0.401	0.578	1.339	0.611
7HV_Molasses	0.654	0.524	0.392	0.572	1.308	0.591
15HV_Milk	0.667	0.532	0.405	0.580	1.334	0.625
15HV_Molasses	0.657	0.526	0.402	0.574	1.314	0.623
32HV_Milk	0.665	0.534	0.410	0.583	1.330	0.640
32HV_Molasses	0.663	0.530	0.413	0.578	1.326	0.659

**Table 2:** ^13^C NMR integrated signals and parameter *D*.

Samples	Monomer Mole Fraction ^a^		Diad Mole Fraction ^b^				*D* Parameter
	*F*_B_	*F*_V_	*F*_BB_	*F*_BV_	*F*_VB_	*F*_VV_	
7HV_Milk	0.934	0.066	0.870	0.064	0.055	0.011	2.80
7HV_Molasses	0.931	0.069	0.831	0.081	0.077	0.011	1.42
15HV_Milk	0.853	0.147	0.862	0.059	0.056	0.024	6.32
15HV_Molasses	0.851	0.149	0.786	0.096	0.089	0.029	2.67
32HV_Milk	0.682	0.318	0.450	0.222	0.213	0.115	1.10
32HV_Molasses	0.683	0.317	0.449	0.223	0.213	0.114	1.08

^a^ Determined from ^1^H NMR from -CH_3_ resonance peaks. ^b^ Determined from ^13^C NMR from -C=O resonance peaks.

**Table 3:** Mechanical properties of PHBV film with varied 3HV content.

Samples	Modulus *E* (MPa)	Tensile Strength (MPa)	Elongation at Break (%)
**7HV_Milk**	1277 ± 39	18.1 ± 0.3	4.3 ± 2.5
**7HV_Molasses**	1129 ± 51	16.5 ± 0.4	4.1 ± 2.4
**15HV_Milk**	920 ± 53	17.0 ± 0.4	8.1 ± 3.2
**15HV_Molasses**	823 ± 47	16.1 ± 0.4	15.6 ± 3.1
**32HV_Milk**	312 ± 59	7.1 ± 0.6	95.1 ± 8.1
**32HV_Molasses**	230 ± 51	5.0 ± 0.5	102.4 ± 7.1

**Table 4:** Optimized electrospinning condition for PHBV samples.

Samples	Solution Concentration (% *w*/*v*)	Applied Voltage (kV)	Feeding Rate (mL/h)	Fiber Diameter (µm)
7HV_Milk	10	15	2	2.14 ± 0.27
7HV_Molasses	10	15	2	2.49 ± 0.24
15HV_Milk	15	15	2	2.34 ± 0.30
15HV_Molasses	15	15	2	2.29 ± 0.35
32HV_Milk	20	15	2	2.91 ± 0.37
32HV_Molasses	20	15	2	2.70 ± 0.43

## Data Availability

Data are contained within the article and supplementary materials.

## Supplementary Materials

The following supporting information can be downloaded at: https://www.mdpi.com/article/10.3390/ijms26010180/s1.

## References

[1] Steinbüchel A., Valentin H.E. (1995). Diversity of Bacterial Polyhydroxyalkanoic Acids. FEMS Microbiol. Lett..

[2] Steinbüchel A., Füchtenbusch B. (1998). Bacterial and Other Biological Systems for Polyester Production. Trends Biotechnol..

[3] Acharjee S.A., Bharali P., Gogoi B., Sorhie V., Walling B., Alemtoshi (2023). PHA-Based Bioplastic: A Potential Alternative to Address Microplastic Pollution. Water Air Soil. Pollut..

[4] Wang S., Chen W., Xiang H., Yang J., Zhou Z., Zhu M. (2016). Modification and Potential Application of Short-Chain-Length Polyhydroxyalkanoate (SCL-PHA). Polymers.

[5] Abbasi M., Pokhrel D., Coats E.R., Guho N.M., McDonald A.G. (2022). Effect of 3-Hydroxyvalerate Content on Thermal, Mechanical, and Rheological Properties of Poly(3-Hydroxybutyrate-Co-3-Hydroxyvalerate) Biopolymers Produced from Fermented Dairy Manure. Polymers.

[6] Policastro G., Panico A., Fabbricino M. (2021). Improving Biological Production of Poly(3-Hydroxybutyrate-Co-3-Hydroxyvalerate) (PHBV) Co-Polymer: A Critical Review. Rev. Environ. Sci. Biotechnol..

[7] Urtuvia V., Maturana N., Peña C., Díaz-Barrera A. (2020). Accumulation of Poly(3-Hydroxybutyrate-Co-3-Hydroxyvalerate) by Azotobacter Vinelandii with Different 3HV Fraction in Shake Flasks and Bioreactor. Bioprocess Biosyst. Eng..

[8] Berezina N., Yada B. (2016). Improvement of the Poly(3-Hydroxybutyrate-Co-3-Hydroxyvalerate) (PHBV) Production by Dual Feeding with Levulinic Acid and Sodium Propionate in Cupriavidus Necator. N. Biotechnol..

[9] Aramvash A., Hajizadeh-Turchi S., Moazzeni-zavareh F., Gholami-Banadkuki N., Malek-sabet N., Akbari-Shahabi Z. (2016). Effective Enhancement of Hydroxyvalerate Content of PHBV in Cupriavidus Necator and Its Characterization. Int. J. Biol. Macromol..

[10] Ganzeveld K.J., van Hagen A., van Agteren M.H., de Koning W., Uiterkamp A.J.S. (1999). Upgrading of Organic Waste: Production of the Copolymer Poly-3-Hydroxybutyrate-Co-Valerate by Ralstonia Eutrophus with Organic Waste as Sole Carbon Source. J. Clean. Prod..

[11] Serafim L.S., Lemos P.C., Albuquerque M.G.E., Reis M.A.M. (2008). Strategies for PHA Production by Mixed Cultures and Renewable Waste Materials. Appl. Microbiol. Biotechnol..

[12] Wang J., Liu S., Huang J., Qu Z. (2021). A Review on Polyhydroxyalkanoate Production from Agricultural Waste Biomass: Development, Advances, Circular Approach, and Challenges. Bioresour. Technol..

[13] Chanasit W., Bunkaew K. (2023). Inexpensive Production of Poly (3-hydroxybutyrate-co-3-hydroxyvalerate) from Bacillus Megaterium PP-10 Using Pineapple Peel Waste. ASEAN J. Sci. Technol. Rep..

[14] Gheibi A., Khoshnevisan K., Ketabchi N., Derakhshan M.A., Babadi A.A. (2016). Application of Electrospun Nanofibrous PHBV Scaffold in Neural Graft and Regeneration: A Mini-Review. Nanomed. Res. J..

[15] Karbowniczek J.E., Kaniuk Ł., Berniak K., Gruszczyński A., Stachewicz U. (2021). Enhanced Cells Anchoring to Electrospun Hybrid Scaffolds with PHBV and HA Particles for Bone Tissue Regeneration. Front. Bioeng. Biotechnol..

[16] Kaniuk Ł., Stachewicz U. (2021). Development and Advantages of Biodegradable PHA Polymers Based on Electrospun PHBV Fibers for Tissue Engineering and Other Biomedical Applications. ACS Biomater. Sci. Eng..

[17] Muigano M.N., Anami S.E., Onguso J.M., Mauti G.O. (2024). Optimized Poly(3-Hydroxybutyrate-Co-3-Hydroxyvalerate) (PHBV) Production by Moderately Haloalkaliphilic Bacterium Halomonas Alkalicola Ext. Int. J. Polym. Sci..

[18] Kansiz M., Domínguez-Vidal A., McNaughton D., Lendl B. (2007). Fourier-Transform Infrared (FTIR) Spectroscopy for Monitoring and Determining the Degree of Crystallisation of Polyhydroxyalkanoates (PHAs). Anal. Bioanal. Chem..

[19] Bossu J., Angellier-Coussy H., Totee C., Matos M., Reis M., Guillard V. (2020). Effect of the Molecular Structure of Poly(3-Hydroxybutyrate-Co-3-Hydroxyvalerate) (P(3HB-3HV)) Produced from Mixed Bacterial Cultures on Its Crystallization and Mechanical Properties. Biomacromolecules.

[20] Pramanik N., Das R., Rath T., Kundu P.P. (2014). Microbial Degradation of Linseed Oil-Based Elastomer and Subsequent Accumulation of Poly(3-Hydroxybutyrate-Co-3-Hydroxyvalerate) Copolymer. Appl. Biochem. Biotechnol..

[21] Bayari S., Severcan F. (2005). FTIR Study of Biodegradable Biopolymers: P(3HB), P(3HB-Co-4HB) and P(3HB-Co-3HV). J. Mol. Struct..

[22] Pradhan S., Dikshit P.K., Moholkar V.S. (2018). Production, Ultrasonic Extraction, and Characterization of Poly (3-Hydroxybutyrate) (PHB) Using Bacillus Megaterium and Cupriavidus Necator. Polym. Adv. Technol..

[23] Yu W., Lan C.H., Wang S.J., Fang P.F., Sun Y.M. (2010). Influence of Zinc Oxide Nanoparticles on the Crystallization Behavior of Electrospun Poly(3-Hydroxybutyrate-Co-3-Hydroxyvalerate) Nanofibers. Polymer.

[24] Xu Y., Zou L., Lu H., Wei Y., Hua J., Chen S. (2016). Preparation and Characterization of Electrospun PHBV/PEO Mats: The Role of Solvent and PEO Component. J. Mater. Sci..

[25] Ivanova G., Serafim L.S., Lemos P.C., Ramos A.M., Reis M.A.M., Cabrita E.J. (2009). Influence of Feeding Strategies of Mixed Microbial Cultures on the Chemical Composition and Microstructure of Copolyesters P(3HB-Co-3HV) Analyzed by NMR and Statistical Analysis. Magn. Reson. Chem..

[26] Žagar E., Kržan A., Adamus G., Kowalczuk M. (2006). Sequence Distribution in Microbial Poly(3-Hydroxybutyrate-Co-3-Hydroxyvalerate) Co-Polyesters Determined by NMR and MS. Biomacromolecules.

[27] Montiel-Jarillo G., Morales-Urrea D.A., Contreras E.M., López-Córdoba A., Gómez-Pachón E.Y., Carrera J., Suárez-Ojeda M.E. (2022). Improvement of the Polyhydroxyalkanoates Recovery from Mixed Microbial Cultures Using Sodium Hypochlorite Pre-Treatment Coupled with Solvent Extraction. Polymers.

[28] Cheng H.N., Biswas A., Vermillion K., Melendez-Rodriguez B., Lagaron J.M. (2020). NMR Analysis and Triad Sequence Distributions of Poly(3-Hydroxybutyrate-Co-3-Hydroxyvalerate). Polym. Test..

[29] Yoshie N., Menju H., Sato H., Inoue Y. (1995). Complex Composition Distribution of Poly(3-Hydroxybutyrate-Co-3-Hydroxyvalerate). Macromolecules.

[30] Gunaratne L.M.W.K., Shanks R.A. (2005). Multiple Melting Behaviour of Poly(3-Hydroxybutyrate-Co-Hydroxyvalerate) Using Step-Scan DSC. Eur. Polym. J..

[31] Guho N.M., Pokhrel D., Abbasi M., Mcdonald A.G., Alfaro M., Brinkman C.K., Coats E.R. (2020). Pilot-Scale Production of Poly-3-Hydroxybutyrate-Co-3-Hydroxyvalerate from Fermented Dairy Manure: 1 Process Performance, Polymer Characterization, and Scale-up Implications. Bioresour. Technol..

[32] Tong H.W., Wang M. (2011). Electrospinning of Poly(Hydroxybutyrate-Co-Hydroxyvalerate) Fibrous Scaffolds for Tissue Engineering Applications: Effects of Electrospinning Parameters and Solution Properties. J. Macromol. Sci. Part B Phys..

[33] Tong H.W., Wang M. (2007). Effects of Processing Parameters on the Morphology and Size of Electrospun PHBV Micro- and Nano-Fibers. Key Eng. Mater..

[34] Tong H.-W., Wang M. (2010). Electrospinning of Fibrous Polymer Scaffolds Using Positive Voltage or Negative Voltage: A Comparative Study. Biomed. Mater..

[35] Tong H.W., Wang M., Lu W.W. (2011). Electrospun Poly(Hydroxybutyrate-Co-Hydroxyvalerate) Fibrous Membranes Consisting of Parallel-Aligned Fibers or Cross-Aligned Fibers: Characterization and Biological Evaluation. J. Biomater. Sci. Polym. Ed..

[36] Tong H.W., Wang M., Lu W.W. (2012). Electrospinning and Evaluation of PHBV-Based Tissue Engineering Scaffolds with Different Fibre Diameters, Surface Topography and Compositions. J. Biomater. Sci. Polym. Ed..

[37] Suwantong O., Waleetorncheepsawat S., Sanchavanakit N., Pavasant P., Cheepsunthorn P., Bunaprasert T., Supaphol P. (2007). In Vitro Biocompatibility of Electrospun Poly(3-Hydroxybutyrate) and Poly(3-Hydroxybutyrate-Co-3-Hydroxyvalerate) Fiber Mats. Int. J. Biol. Macromol..

[38] Sombatmankhong K., Sanchavanakit N., Pavasant P., Supaphol P. (2007). Bone Scaffolds from Electrospun Fiber Mats of Poly(3-Hydroxybutyrate), Poly(3-Hydroxybutyrate-Co-3-Hydroxyvalerate) and Their Blend. Polymer.

[39] Kaniuk Ł., Krysiak Z.J., Metwally S., Stachewicz U. (2020). Osteoblasts and Fibroblasts Attachment to Poly(3-Hydroxybutyric Acid-Co-3-Hydrovaleric Acid) (PHBV) Film and Electrospun Scaffolds. Mater. Sci. Eng. C.

[40] Kouhi M., Prabhakaran M.P., Shamanian M., Fathi M., Morshed M., Ramakrishna S. (2015). Electrospun PHBV Nanofibers Containing HA and Bredigite Nanoparticles: Fabrication, Characterization and Evaluation of Mechanical Properties and Bioactivity. Compos. Sci. Technol..

[41] Zhang S., Prabhakaran M.P., Qin X., Ramakrishna S. (2015). Biocomposite Scaffolds for Bone Regeneration: Role of Chitosan and Hydroxyapatite within Poly-3-Hydroxybutyrate-Co-3-Hydroxyvalerate on Mechanical Properties and in Vitro Evaluation. J. Mech. Behav. Biomed. Mater..

[42] Meng W., Kim S.Y., Yuan J., Kim J.C., Kwon O.H., Kawazoe N., Chen G., Ito Y., Kang I.K. (2007). Electrospun PHBV/Collagen Composite Nanofibrous Scaffolds for Tissue Engineering. J. Biomater. Sci. Polym. Ed..

[43] Lee I.S., Kwon O.H., Meng W., Kang I.K., Ito Y. (2004). Nanofabrication of Microbial Polyester by Electrospinning Promotes Cell Attachment. Macromol. Res..

[44] Ito Y., Hasuda H., Kamitakahara M., Ohtsuki C., Tanihara M., Kang I.K., Kwon O.H. (2005). A Composite of Hydroxyapatite with Electrospun Biodegradable Nanofibers as a Tissue Engineering Material. J. Biosci. Bioeng..

[45] Kaniuk Ł., Berniak K., Lichawska-Cieślar A., Jura J., Karbowniczek J.E., Stachewicz U. (2022). Accelerated Wound Closure Rate by Hyaluronic Acid Release from Coated PHBV Electrospun Fiber Scaffolds. J. Drug Deliv. Sci. Technol..

[46] Dinos G.P., Athanassopoulos C.M., Missiri D.A., Giannopoulou P.C., Vlachogiannis I.A., Papadopoulos G.E., Papaioannou D., Kalpaxis D.L. (2016). Chloramphenicol Derivatives as Antibacterial and Anticancer Agents: Historic Problems and Current Solutions. Antibiotics.

[47] Pramanik U., Khamari L., Shekhar S., Mukherjee S. (2020). On the Role of Hydrophobic Interactions between Chloramphenicol and Bovine Pancreatic Trypsin: The Effect of a Strong Electrolyte. Chem. Phys. Lett..

[48] Miller S.I. (2016). Antibiotic Resistance and Regulation of the Gram-Negative Bacterial Outer Membrane Barrier by Host Innate Immune Molecules. mBio.

[49] Lemes A.P., Montanheiro T.L.D.A., Durán N., da Silva A.P. (2019). PHBV/MWCNT films: Hydrophobicity, thermal and mechanical properties as a function of MWCNT concentration. J. Compos. Sci..

[50] Khamplod T., Winterburn J.B., Cartmell S.H. (2022). Electrospun Poly(3-Hydroxybutyrate-Co-3-Hydroxyvalerate) Scaffolds–a Step towards Ligament Repair Applications. Sci. Technol. Adv. Mater..

[51] Rivas M., Del Valle L.J., Rodríguez-Rivero A.M., Turon P., Puiggalí J., Alemán C. (2018). Loading of Antibiotic into biocoated hydroxyapatite nanoparticles: Smart antitumor platforms with regulated release. ACS Biomater. Sci. Eng..

[52] Rosengart A., Cesário M.T., de Almeida M.C.M.D., Raposo R.S., Espert A., de Apodaca E.D., da Fonseca M.M.R. (2015). Efficient P(3HB) Extraction from Burkholderia Sacchari Cells Using Non-Chlorinated Solvents. Biochem. Eng. J..

[53] Barham P.J., Keller A., Otun E.L., Wills H.H., Holmes P.A. (1984). Crystallization and morphology of a bacterial thermoplastic: Poly-3-hydroxybutyrate. J. Mater. Sci..

[54] Jun Z., Hou H., Schaper A., Wendorff J.H., Greiner A. (2003). Poly-L-lactide nanofibers by electrospinning–Influence of solution viscosity and electrical conductivity on fiber diameter and fiber morphology. e-Polymers.

[55] Ol’khov A.A., Staroverova O.V., Gol’dshtrakh M.A., Khvatov A.V., Gumargalieva K.Z., Iordanskii A.L. (2016). Electrospinning of Biodegradable Poly-3-Hydroxybutyrate. Effect of the Characteristics of the Polymer Solution. Russ. J. Phys. Chem. B.

