# Supplementary material for: Characterization and Biomedical Applications of Electrospun PHBV Scaffolds Derived from Organic Residues
Source: Int J Mol Sci. 2024 Dec 28;26(1):180. doi: 10.3390/ijms26010180 (PMC11719612; doi:10.3390/ijms26010180)
Supplement: Supplementary file 1 [file ijms-26-00180-s001.zip › ijms-3361519-supplementary.pdf]

Supplementary Materials

Table S1. Thermal properties of PHBV samples derived from milk and molasses residues.

Samples	Cooling			Second heating					
	T_c (°C)	ΔH_c (J/g)	T_g (°C)	T_{cc} (°C)	ΔH_{cc} (°C)	T_{m1} (°C)	T_{m2} (°C)	ΔH_f (°C)	χ^a (%)
**7HV_Milk**	79.9	58.1	-1.8	50.7	5.1	145.1	158.3	71.1	48.7
**7HV_Molasses**	64.2	9.8	-2	60.8	50.4	132.1	149.5	62.7	42.9
**15HV_Milk**	97.1	66.3	3.8	47.9	0.3	158.2	167.5	70.6	48.3
**15HV_Molasses**	58.5	8.8	1.3	58.6	42.5	145.4	163.8	54.2	35.8
**32HV_Milk**	-	-	-4.2	-	-	-	-	-	-
**32HV_Molasses**	-	-	-5.7	-	-	-	-	-	-

[a] Percentage of crystallinity calculated from second heating cycle using equation (1).

Table S2. Degradation properties of PHBV samples.

Sample	$T_{5\%}$ (°C)	$T_{50\%}$ (°C)	$T_{90\%}$ (°C)	Residue at 310 °C (wt%)
7HV_Milk	232	263	274	2.1
7HV_Molasses	243	270	283	1.4
15HV_Milk	269	286	293	3.6
15HV_Molasses	279	298	306	1.8
32HV_Milk	255	287	294	5.3
32HV_Molasses	262	289	299	1.5
